# Resource utilization and outcome at a university versus a community teaching hospital in tPA treated stroke patients: a retrospective cohort study

**DOI:** 10.1186/1472-6963-10-44

**Published:** 2010-02-19

**Authors:** Angela F Caveney, Robert Silbergleit, Shirley Frederiksen, William J Meurer, Susan L Hickenbottom, Rodney W Smith, Phillip A Scott

**Affiliations:** 1 Department of Psychiatry, University of Michigan, Commonwealth Blvd, Ann Arbor, MI, USA; 2 Department of Emergency Medicine, University of Michigan, University of Michigan, Medical Center Drive, Ann Arbor, MI, USA; 3 Department of Neurology, St Joseph Mercy Health System, McAuley Drive, Ypsilanti, MI, USA; 4 Department of Emergency Medicine, St Joseph Mercy Health System, McAuley Drive, Ypsilanti, MI, USA

## Abstract

**Background:**

Comparing patterns of resource utilization between hospitals is often complicated by biases in community and patient populations. Stroke patients treated with tissue plasminogen activator (tPA) provide a particularly homogenous population for comparison because of strict eligibility criteria for treatment. We tested whether resource utilization would be similar in this homogenous population between two hospitals located in a single Midwestern US community by comparing use of diagnostic testing and associated outcomes following treatment with t-PA.

**Methods:**

Medical records from 206 consecutive intravenous t-PA-treated stroke patients from two teaching hospitals (one university, one community-based) were reviewed. Patient demographics, clinical characteristics and outcome were analyzed, as were the frequency of use of CT, MRI, MRA, echocardiography, angiography, and EEG.

**Results:**

Seventy-nine and 127 stroke patients received t-PA at the university and community hospitals, respectively. The two patient populations were demographically similar. There were no differences in stroke severity. All outcomes were similar at both hospitals. Utilization of CT scans, and non-invasive carotid and cardiac imaging studies were similar at both hospitals; however, brain MR, TEE, and catheter angiography were used more frequently at the university hospital. EEG was obtained more often at the community hospital.

**Conclusions:**

Utilization of advanced brain imaging and invasive diagnostic testing was greater at the university hospital, but was not associated with improved clinical outcomes. This could not be explained on the basis of stroke severity or patient characteristics. This variation of practice suggests substantial opportunities exist to reduce costs and improve efficiency of diagnostic resource use as well as reduce patient exposure to risk from diagnostic procedures.

## Background

Stroke is a leading cause of death and disability in the United States as well as throughout developed countries world-wide. Every year, there are approximately 700,000 new or recurring strokes in the United States, with one in 16 deaths in 2004 being attributed to stroke [1]. The overall direct and indirect costs associated with stroke in the United States alone are estimated at $62.7 billion in 2007 [1]. Of this, approximately 18 billion dollars is expected to go toward direct in-hospital costs, not including physician fees or medications [1].

Diringer and colleagues [2] examined the breakdown of hospital costs in 191 individuals presenting with acute ischemic stroke to a single tertiary care academic hospital. These researchers estimated 50% of costs were attributed to bed charges (16% ICU, 34% ward). An additional 19% (approximately $3.5 billion in 2007) were associated with diagnostic tests. Reed [3] argued that different types of hospitals should be examined separately as costs can differ dramatically between facilities. For example, these researchers found that costs in teaching community hospitals were 10-30% greater than those at non-teaching community hospitals. Furthermore, they estimated that, overall, community hospital costs were approximately 10-20% lower than academic medical center costs.

Variability in spending by hospital type is likely to be a reflection of variability in practice, particularly with regard to the use of diagnostic tests. Practice variability is common for all diagnoses, and studying variability is often used as an approach to improving quality of care or to reducing expense. Evaluation of practice variability is, however, confounded by local referral patterns or differences in the patient populations served by different doctors and different facilities. Variability in patient populations including differences in age, disease severity, acuity, and comorbidities are potential confounders that make examining practice variability even more difficult. In stroke care in particular, differences in patients' ages, comorbidities, and stroke severity may distinguish patients treated in tertiary care hospitals from those treated in community hospitals.

Many studies of economic burden associated with stroke [4-6] group all types of stroke (including subarachnoid hemorrhage, intracerebral hemorrhage and transient ischemic attacks) with ischemic stroke. Reed and colleagues [3] argued that it is important to break stroke subtypes down by category as different types of strokes would be associated with different lengths of stay (LOS) and different hospital procedures, leading to significantly differing costs. These researchers calculated that, in 1998, the average per incident inpatient costs was approximately $5837 specifically for ischemic stroke.

In our community, stroke patients from a tri-county area are treated in two large tertiary care teaching hospitals. Both hospitals draw from the same population base, and are served by the same emergency medical system (EMS). By virtue of their condition and the imposition of a narrow treatment window, acute stroke patients treated with t-PA are unlikely to be influenced by differences in hospital referral patterns, and are likely similar with regard to stroke severity, age, and comorbid conditions. We therefore used this sample of patients as a natural experiment to examine variability in clinical practice.

The objectives of this study were to determine the extent to which inter-hospital variability in practice exists and whether variability in practice is associated with stroke outcomes. We assessed whether resource utilization would be similar in such a homogenous population between two hospitals located in a single Midwestern US community by comparing use of diagnostic testing and associated outcomes following treatment with t-PA.

## Methods

Data were collected as part of a larger retrospective, observational study evaluating the safety and effectiveness of t-PA use in stroke patients [7]. Study methodology is summarized below. The study was approved by the Institutional Review Boards at the participating institutions.

### Study Design and Setting

Medical records from 206 consecutive patients with acute ischemic stroke (AIS) treated with IV t-PA in the emergency departments of two large tertiary care teaching hospitals (one university hospital staffed by medical school faculty, one community-based staffed by privately practicing physicians) in Southeastern Michigan were reviewed. The University Hospital and the Community Hospital are located within 6 miles of each other. The two hospitals are similar in size (792 versus 529 beds, respectively) and adult patient volume (approximately 51,000 versus 65,000 ED visits per year in 2004), and are the only tertiary care centers within a 30 mile radius. The county in which these hospitals are located has a population of approximately 300,000 and includes urban, suburban, and rural areas. Both hospitals have residency training programs in emergency medicine and several other specialties. The community hospital does not have training programs in either neurology or neurosurgery.

All patients included in this sample presented to the emergency departments within three hours of onset of an ischemic stroke. All were administered intravenous t-PA. All aspects of care were at the discretion of the treating physicians based on hospital standards.

### Case Identification

All patients with AIS treated with t-PA between January 1, 1996 and January 1, 2005 were identified via four methods (billing data, pharmacy logs, hospital stroke registries and Paul Coverdell National Acute Stroke registry data) at each site.

### Data Collection

Four clinically trained, National Institutes of Health Stroke Scale (NIHSS) certified, reviewers (3 RNs, 1 Clinical Ph.D.) abstracted data for each confirmed case from the paper and/or electronic medical record. Reviewers were unaware of the hypothesis or analysis presented here at the time of the chart review. Coding uncertainties were documented and resolved by consensus of the reviewers with an investigator (PAS or RS). The first 10% of records were dual abstracted as well as an additional 10% random sample of the remaining records to assess inter-rater agreement.

Information recorded included, patient demographics, clinical characteristics, emergency department and inpatient resource utilization. Specifically, the frequency and use of procedures including computed tomography (CT), magnetic resonance imaging (MRI) and magnetic resonance angiography (MRA) of the brain as well as transthoracic (TTE) and transesophageal (TEE) echocardiography, standard angiography, and EEG were recorded. Patient oriented outcomes obtained included presence of any infarct-related hemorrhage, clinical improvement, hospital length of stay, discharge location, estimated mRS at discharge and mortality.

Total hospital LOS was defined as the initial period of hospitalization from emergency department presentation to discharge from the acute care hospitalization. Time in inhospital rehabilitation was not included.

### Statistical Analyses

Descriptive statistics are presented. Student's *t*-test (continuous measures) or chi square (proportions) were used to compare resource utilization and outcomes between hospitals. All *P *values were 2-tailed and considered significant when less than 0.05.

## Results

Sixty-two percent (127) of patients in the study presented to the community hospital. Baseline demographics and clinical characteristics of the treated population in the study are presented in Table 1. Most baseline patient characteristics, including risk factors for stroke, were similar between the two hospitals, however, functional deficits (as measured by an estimated modified Rankin Scale rating) prior to the onset of stroke were more common in patients treated at the community hospital as compared to the university (34% v. 16%, p < 0.01). There was no evidence of a difference in stroke severity based on NIHSS (p > 0.86). The vast majority of patients had moderate to severe strokes, with only 5% falling in the mild range (NIHSS ≤ 5). There was also no difference between hospitals in the total rate of any intracranial hemorrhage occurring during the index admission (p = .712).

**Table 1:** Patient Baseline Demographic and Clinical Characteristics by Hospital

		Total	Community	University	*P*
		(N = 206)	(N = 127)	(N = 79)	
Age; *mean ± stdev*		68 ± 15.8	69 ± 15.8	66 ± 15.9	.147
Sex, male; *n, (%)*		111 (54)	67 (53)	44 (56)	.681
Race, Caucasian; *n, (%)*		179 (87)	109 (86)	70 (89)	.358
					
Pre-Stroke mRS; *Median (IQR)*		1 (0-2)	1 (0-2)	0 (0-1)	.001
Pre-tx NIHSS;*Median (IQR)*		13 (8-17)	13 (8-17)	14 (8-17)	.867
					
Intracranial Hemorrhage**; *n, (%)*		37 (18)	24 (19)	13 (16)	.712
					
t-PA time*; *mean ± stdev*		155 ± 33	157 ± 31	151 ± 36	.185
					
Baseline stroke risk factors		*n, (%)*			
Prior Stroke		34 (17)	23 (18)	11 (14)	.431
Diabetes		44 (21)	28 (22)	16 (20)	.760
Hypertension		142 (69)	92 (72)	50 (63)	.168
High cholesterol		66 (32)	41 (32)	25 (32)	.924
Hx of coronary artery disease		65 (32)	41 (32)	24 (30)	.775
Hx of congestive heart failure		20 (10)	13 (10)	7 (9)	.746
Hx of atrial fibrillation		58 (28)	34 (27)	24 (30)	.576
Smoking w/in 1 year		51 (25)	31 (24)	20 (25)	.883

*Time, in minutes, from stroke onset to tPA bolus administration.

**Defined as any intracranial hemorrhage at any time point during the index hospitalization.

Abbreviations: mRS = modified estimated Rankin Scale; NIHSS = estimated National Institutes of Health Stroke Scale.

### In-Hospital Resource Utilization

Utilization of CT scans, as well as non-invasive (ultrasound) carotid and transthoracic cardiac imaging were similar at both hospitals. Brain MRI and MRA, as well as TEE, and catheter angiography, were used more often at the university hospital. EEG was obtained more often at the community hospital (Table 2).

**Table 2:** Patient Outcomes and Acute Hospitalization Resource Utilization by Hospital

		Total	Community	University	*P*
		**(N = 206)**	**(N = 127)**	**(N = 79)**	

Tests Performed			*n, (%)*		

CT brain		206 (100)	127 (100)	79 (100)	1.000

Follow up CT brain		152 (74)	93 (73)	59 (75)	.817

MRI brain		49 (24)	21 (17)	28 (35)	.002

MRA brain		35 (17)	11 (9)	24 (30)	<.001

MRA neck		38 (18)	20 (16)	18 (23)	.205

Carotid Doppler		114 (55)	71 (56)	43 (54)	.836

Angiography		17 (8)	6 (4)	11 (13)	.020

Transthoracic Echo		58 (28)	31 (24)	27 (34)	.130

Transesophageal Echo		69 (33)	32 (25)	37 (47)	.001

EEG		15 (7)	13 (10)	2 (3)	.039

					

Outcomes			*mean ± stdev*		

LOS - all (days)		6.75 ± 4.6	6.33 ± 4.3	7.43 ± 5.0	.096

LOS - surviving (days)		6.91 ± 4.7	6.42 ± 4.4	7.65 ± 4.9	.084

Improved from admission		154 (75%)	90 (71%)	64 (81%)	.137

					

Discharge destination/status			*n, (%)*		.302

Home		79 (38)	42 (33)	37 (47)	

Inpatient rehab		60 (29)	39 (31)	21 (27)	

Other acute care hosp		4 (2)	2 (2)	2 (3)	

Nursing home/assist living		34 (17)	23 (18)	11 (14)	

Death		29 (13)	21 (17)	8 (10)	

Abbreviations: CT = computed tomography; MRI = magnetic resonance imaging; MRA = magnetic resonance angiography; EEG = electroencephalogram; LOS = length of stay.

### Survival, Length of Stay, Discharge Location and Outcome

There was no significant difference in in-hospital mortality, or survival at one year post-treatment (Table 2). In the community hospital, 83% (106 of 127) patients survived to discharge and 70% (89 of 127) to one year. In the academic hospital 90% (71 of 79) patients survived to discharge and 75% (59 of 79) to one year.

Hospital LOS was similar between hospitals with an average of 6.8 days for all patients (Table 2). There were also no between-hospital differences for LOS when only patients who survived to discharge were considered. Discharge destination and functional status did not differ. Abstractors' estimates of patients' functional status at discharge compared to that at admission (i.e., improved vs. unchanged or worse) also did not differ by hospital (p = .137).

### Data Quality

Double reviews were completed on over 35% of the charts. Overall raw agreement for all chart abstraction items including binary, ordinal, continuous, and nominal categorical variables was excellent at 97% (95% CI: 0.973 - 0.976). For the binary and ordinal chart abstraction items the kappa statistic ranged from 0.49 to 0.96 for pre-determined critical variables.

## Discussion

Consistent with Reed's findings [3], these data confirm the existence of practice variability in stroke care between a community hospital and a university hospital, even in a highly homogenous population of stroke patients treated with thrombolytic therapy. There was minimal variability in what can be described as "basic" diagnostic tests such as CT imaging, carotid Doppler imaging, and transthoracic echocardiography. Rather, care at the university hospital differed from that at the community hospital with increased frequency of use of what can be described as "advanced" imaging tests: MRI, MRA, TEE, and catheter angiography. In addition, there was increased use of EEG at the community hospital, an uncommon post-stroke diagnostic modality.

Identification of practice variability does not provide direct evidence of comparative effectiveness across either populations or in specific individuals. That is, we have no way of knowing from these data whether more or fewer diagnostic procedures indicate better or worse care. On the other hand, it makes sense to look for variability in care, especially when it involves expensive modalities and does not have obvious impact on outcomes, as these are potentially high yield targets for prospective comparative effectiveness research and reductions in health care expenditures. Practice variability had no apparent association with clinical outcome in these data. The most obvious implication of this information is that the increased use of advanced imaging at the university hospital likely added to the costs of patient care without adding to patient benefit. A possible alternative explanation is that there may be benefit to the increased use of advanced imaging that was simply too small to detect in these data. Indeed, outcomes were consistently numerically superior in patients treated at the university hospital, but these did not reach statistical significance on any outcome measure.

These associative data do not say anything about the use of advanced imaging in selected patients. Indeed it was available and utilized for some patients at both hospitals, but advanced imaging was apparently used more selectively in the community setting and more liberally in the university hospital. This difference may reflect the increased amount of resident decision making in diagnostic ordering at the university hospital or possible differences over the course of the study in the availability or access to advanced neurodiagnostic tests between the two facilities.

It is harder to speculate on why there is higher usage of EEGs at the community hospital. A detailed examination of the 13 individuals receiving EEG at the community hospital revealed that the indications given for the study were suspected seizures in three of these patients, five others had complicated presentations including significant psychiatric illness in some cases, and an additional three were comatose suggesting the EEG may have been used for prognostic purposes. We cannot entirely exclude the possibility the increased use of EEG may reflect collegial pressures to order and utilize intradepartmental or intra-practice services in this private practice environment.

Another potential explanation of the variation in test ordering practices at the two hospitals might be related to differing degrees of utilization review and differing effectiveness of cost containment initiatives. The majority of patients presenting to both hospitals were over the age of 65 and thus, likely paid by Medicare via a Diagnosis-Related Group (DRG) reimbursement system. Therefore, it would be expected that the reimbursement rates would be similar between the two hospitals. It is possible, however, that the community hospital is more conscious of fiscal issues and thus has become more efficient at monitoring and controlling resource use.

Significant strengths of this study design are related to the homogeneity of patient groups examined as well as the similarities between the two hospitals allowing a more true comparison between factors associated with hospital-specific practice differences without the usual confounders of differing patient characteristics or even geographic differences between hospitals and patients. Another strength is that the chart reviewers were all clinical personnel with stroke experience, rigorously trained and provided with clearly defined data definitions and rules for abstraction. This is evidenced by the high interrater agreement found.

Limitations of this study include those commonly associated with retrospective chart review. Though these charts were often particularly detailed due to the use of thrombolytics, there remain unavoidable problems with data abstraction. Additionally, the outcome variable of patient status at discharge compared to that at admission was subjective and based on the abstractors' estimates derived from information contained within the medical chart. Although the patient populations identified in the two hospitals appear highly homogenous, and EMS protocols dictate delivery of stroke patients to the nearest facility independent of other factors, there could still be unnoticed differences that reflect EMS delivery of some patients to one hospital rather than the other. Finally, although a strength in terms of comparison of two hospitals' responses to a relatively homogenous patient group, the sole use of patients presenting with stroke who were eligible for t-PA potentially limits the generalizability and conclusions that can be made about hospital resource utilization in other patient populations.

## Conclusion

In summary, practice variability was found between a community and a university hospital in the care of AIS patients treated with t-PA. There was no apparent association with clinical outcome. These data suggest that more selective use of advanced imaging in this university hospital, and of EEGs in this community hospital, offers an opportunity for reducing costs and improving efficiency in the care of stroke patients. Realization of these improvements may require examining the effects of organizational culture and fiscal incentives. Leveraging the technique of examining homogenous patient populations may allow identification of systematic inefficiencies in other disease conditions or healthcare facilities.

## Competing interests

The authors declare that they have no competing interests.

## Authors' contributions

RS, SF and PAS conceived the study and participated in study design. AFC and SF participated in study data collection. AFC performed statistical analysis. All authors assisted with drafting, review and approval of the final manuscript.

## Pre-publication history

The pre-publication history for this paper can be accessed here:

http://www.biomedcentral.com/1472-6963/10/44/prepub
